# Measles Outbreak in a Rural Population in Bahar District, Hamadan Province, West of Iran in 2018

**DOI:** 10.34172/jrhs.2020.05

**Published:** 2020-02-27

**Authors:** Manoochehr Karami, Salman Khazaei, Seyed Mohsen Zahraei, Talat Mokhtari Azad, Ali Zahiri, Ali Reza Moradi, Jalal Bathaei, Masoumeh Javaheri, Jalaleddin Amiri

**Affiliations:** ^1^Department of Epidemiology, School of Public Health, Hamadan University of Medical Sciences, Hamadan, Iran; ^2^Modeling of Non-communicable Diseases Research Center, Hamadan University of Medical Sciences, Hamadan, Iran; ^3^Research Center for Health Sciences, Hamadan University of Medical Sciences, Hamadan, Iran; ^4^Center for Communicable Diseases Control, Ministry of Health and Medical Education, Tehran, Iran; ^5^Deputy of Health, Hamadan University of Medical Sciences, Hamadan, Iran; ^6^Department of Pediatrics, School of Medicine, Hamadan University of Medical Sciences, Hamadan, Iran

**Keywords:** Seroepidemiologic studies, Measles, Immunity

## Abstract

**Background:** Measles is in elimination phase in Iran. Following occurrence of two cases of measles in two children under six years old with the history of measles immunization in one of the villages affiliated to Bahar District, west of Hamadan Province, northwest of Iran, this study was conducted to determine the immunity status of people living in the village affected by the measles outbreak in spring of 2018.

**Study design:** A cross-sectional (descriptive-analytical) study.

**Methods:** Overall, 272 populations of 0-40 yr old in the village affected by outbreak were enrolled. Multistage sampling was used for choosing participants. The data collection tool was a researcher-made checklist used to collect epidemiological data including demographic characteristics and vaccination status. Blood samples were taken from them and sent to the measles reference laboratory for IgG testing. The amount of optical density (OD) greater than 0.2 was considered as positive and less than 0.1 negative, respectively.

**Results:** The mean age of the study population was 17.4 ±11.8 yr and the sex ratio was almost the same. Levels of antibodies against measles were positive in 63.24%, intermediate in 12.5% and negative in 24.26%. There was no significant difference between the immunity status of the subjects with gender (P=0.236) and age group (P=0.113). Pearson correlation results showed that in males there was a significant positive correlation between the age of the participants and the serum IgG level (r = 0.26, P=0.003).

**Conclusion:** Measles immunity in communities is not sufficient to prevent outbreaks and small epidemics, and it is recommended that periodically, serological assessments carried out at community level and especially at high-risk groups.

## Introduction

Measles is a highly contagious, acute viral disease and can result in symptoms and complications such as fever, rash, conjunctivitis, diarrhea, pneumonia, encephalitis, and sometimes death ^1^. Despite the availability of a safe and effective measles vaccine, the disease continues to be a major health problem worldwide. Globally measles deaths have decreased by 73% from more than 536,000 deaths in 2000 to about 142,000 deaths in 2018. However, more than 95% of measles deaths occur in countries with low per capita income and poor health infrastructure ^2^. Nearly 10% of under-5 deaths in developing countries are attributable to measles ^2,3^.

Vaccine failure and vaccination problems can be two reasons for children to be susceptible to measles. In general, the gradual accumulation of disease susceptible individuals due to inadequate vaccination or failure to respond to immunization can predispose to measles outbreaks ^4^. Approximately 95% of children who receive a measles vaccine at the age of 12 months develop antibodies against measles in their bodies. However, children receiving two doses of vaccine had a higher level of protection^5,6^.

The national immunization program policy follows the recommendations of WHO for developing countries, and two doses of MMR vaccine are inoculated at one year age and 18 months. Prior to the implementation of the mass immunization program in Iran, measles cases have been reported up to 150,000 in non-epidemic years and 500,000 in epidemic years. Information from National Notifiable Diseases Surveillance System (NNDSS) has shown a sharp decrease in the incidence of the disease in country ^6^.

Numerous studies have investigated the role of some factors such as age, geographic area, history of measles vaccination, and migration history on immunity of population^7-11^, but few studies have examined the efficacy of measles vaccination and its predictors. The study carried out in the province shows almost complete coverage of measles vaccination at the provincial level ^12^. Despite relatively complete vaccination against measles in accordance with the national immunization program, but there are still occurs cases of measles, including in Hamadan Province.

In July 2018, two cases of measles in two children under the age of six with the history of measles immunization was occurred in one of the suburbs of Bahar district. Since measles is in the elimination phase in Iran and even occurrence one case of measles can also be considered an outbreak, therefore, the present study was aimed to determine the measles immunity status of people living in the village affected by the measles outbreak in 2018.

## Methods

The present cross-sectional (descriptive-analytical) study was performed on 272 populations of 0-40 yr old in the village affected by outbreak in Bahar District in 2018. Bahar District located in the west of Hamadan Province, northwest Iran. At the 2016 census, the county's population was 119,082.

The justification for choosing this age group was that, the effectiveness of measles vaccine also was important for us. Therefore we assessed populations with history of measles vaccination. Mass immunization with live measles vaccine in Iran was introduce in 1967, therefore above 50 yr age population during this study did not have history of measles vaccination according routine national immunization program. In 2003, through MR campaign 5-25 yr populations were vaccinated, that they were in 20-40 yr age group in 2018. Based on the foregoing, we decided assess immunity status of populations of 0-40 yr old in the mentioned affected village by outbreak.

Multistage sampling was used for choosing participants. Since the outbreak affected village had 1,005 households (3,328 populations), according to the pre-estimated required sample size, the sampling interval was defined and systematically identified eligible households (Systematic sampling). By referring to households, sampling and completing a checklist were carried out if a person was identified in the pre-defined age and sex groups (Stratified sampling).

The data collection tool was a researcher-developed information sheet used to collect epidemiological data including demographic characteristics and vaccination status. The questionnaire was completed by trained public health experts and two laboratory staff were selected for sampling. In the absence of a household, it would eventually be referred to the household twice, and if not available, the next household would be replaced.

Blood samples were taken from the eligible participation by qualified personnel. Serum samples were stored in a refrigerator (2-8 °C), and in accordance with the principles of cold chain sent to the measles reference laboratory located in the Faculty of Health, Tehran University of Medical Sciences for IgG testing. Measles virus-specific IgG antibodies were detected using an enzyme immunoassay (EIA) kit (DIAsource, Belgium). The amount of optical density (OD) greater than 0.2 was considered as deemed positive, 0.1-0.2 as borderline and less than 0.1 negative, respectively ^13^.

Chi-square test was used to determine the relationship between the gender and age of subjects and the immunity status (immune/non-immune). The correlation between the IgG level and the age of the subjects was evaluated by Pearson correlation coefficient. Logistic regression was conducted to estimate association between subject's age and gender and their measles immunity. Data were analyzed using Stata 14 software at 5% error level.

## Results

Overall, 272 participants, were included in the study. The gender distribution was relatively equal (female to male ratio=1.03). In terms of age, 31.25% were in the 5-15 yr age group and the mean age of the study population was 17.4 ±11.89 yr. Overall, IgG against measles was positive in 63.24% of the subjects, and there was no significant difference between gender (*P* =0.236) and age of participants (*P* =0.113) with IgG level (Table 1).

**Table 1:** Immunity status against measles in levels of gender and age group

**Variables**					**Sero-positive**				***P*** **value**
	**IgG**		**Sero-negative**		**Borderline or equivocal**		**Deemed positive**		
	**Mean**	**SD**	**Number**	**Percent**	**Number**	**Percent**	**Number**	**Percent**	
Gender									0.236
Male	0.29	0.33	29	21.64	21	15.67	84	62.70	
Female	0.27	0.30	37	26.81	13	9.42	88	63.77	
Age group (yr)									0.113
0-4	0.29	0.25	13	23.21	5	8.93	38	67.86	
5-14	0.23	0.22	23	27.06	12	14.12	50	58.82	
15-29	0.28	0.39	19	26.39	14	19.44	39	54.17	
30-40	0.35	0.37	11	18.64	3	5.08	45	76.27	
Total	0.28	0.22	66	24.26	34	12.50	172	63.24	

**Figure 1:**
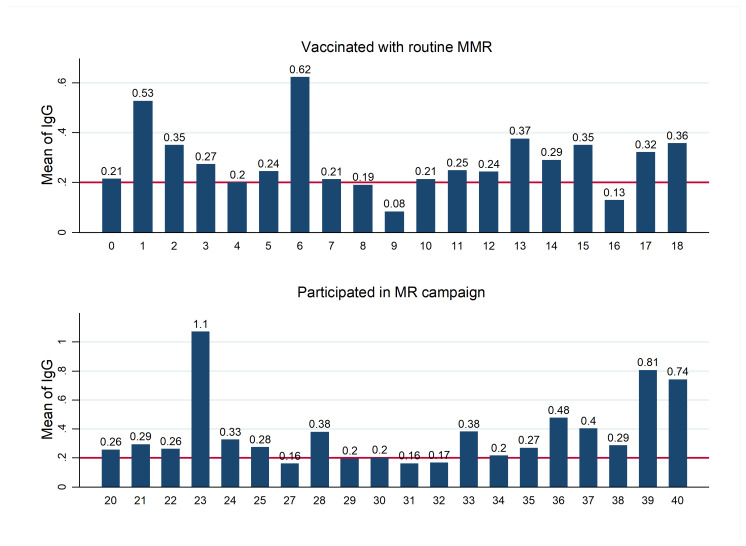
The mean of IgG in different age levels of participants

The mean of IgG in different age levels of the participants is shown in Figure 1, as shown there is no apparent increase or decrease in the mean of IgG according to participant's age. Compared visually, people immunized through MR vaccine campaigns have higher levels of immunity.

**Figure 2:**
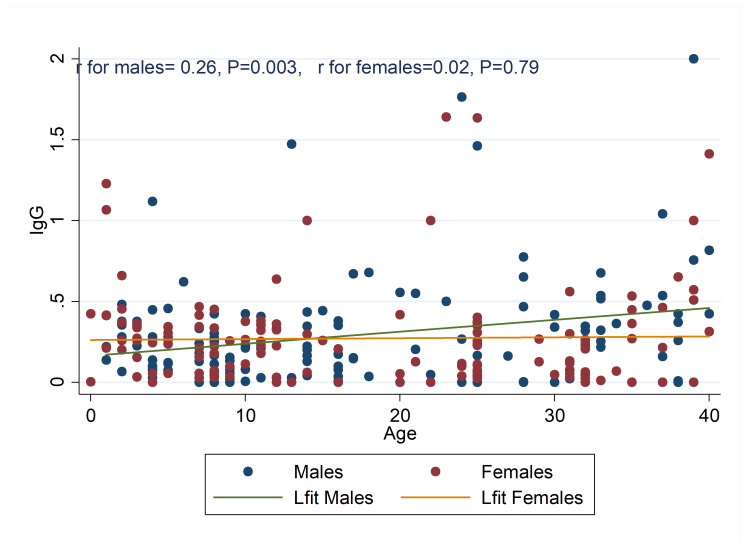
Correlation between the age of the participants and the amount of IgG

Correlation between the age of the participants and the amount of IgG against measles by gender. Against females, in males, there was a significant positive correlation between the age of the participants and the serum IgG level (r = 0.26, *P* = 0.003) (Figure 2).

**Figure 3:**
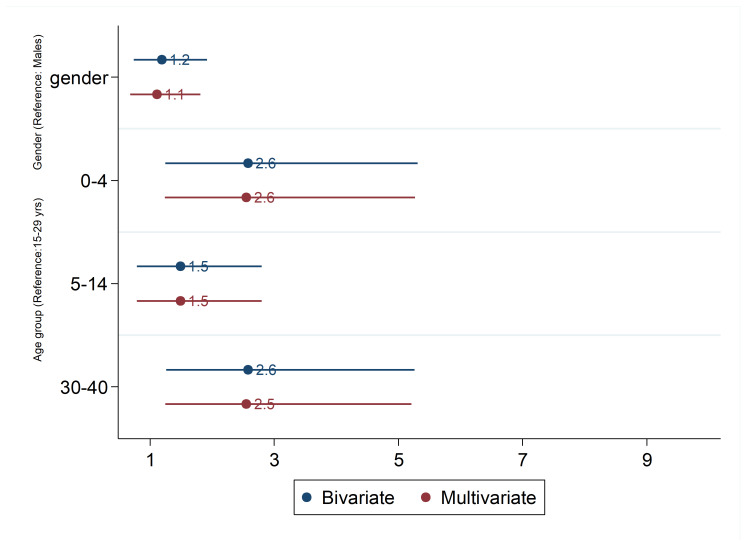
Magnitude of association between measles immunity level and gender and age

Results of the logistic regression have been showed in Figure 3. After adjusting gender, odds of been immune against measles was 2.6 among 0-4 yr age groups (OR=2.55, 95% CI: 1.24, 5.27; *P* =0.011) and 2.5 among 30-40 yr age group (OR=3.54, 95% CI: 1.25, 5.21; *P* =0.010) compared with participations in 15-29 years.

## Discussion

To the best of our knowledge, we assessed the measles seroprevalence in the affected region by measles outbreak in Bahar district, Hamadan, Iran. We examined the level of measles immunity at ages 0-40 yr, separately, in order to predict the at-risk age groups as well as determine the level of measles immunity in a vaccinated population in an area of Iran as a country in the measles elimination step.

In our study, 272 populations, 1-40 yr old, were screened for the presence of anti-measles antibodies. Of them, 172 (63.24%), had positive IgG against measles. Results of the cross-sectional study on 1368 children aged 30–54 months on south-east of Iran showed the 93.7% prevalence rate of anti-measles IgG ^14^. Similar result was reached in another study in south-west of Iran on 900 populations under 25 yr which indicates 91.2% immunity against measles ^15^. In Pakistan as a neighborhood country and endemic country for measles disease, 93.5% of children of 1 to 10 yr of age were immune against measles ^16^. Results of the 16-year review of 68 measles seroprevalence studies showed that measles Seroprevalence was ranged from 60.8% to 95.9% ^17^. The difference in the age range of studies population (infants, children, adolescents and adults or combinations of them) and differences in antibody testing can partially justify these differences. Validation of vaccination coverage should be approved by serological surveys in defining the real protection of the community against measles outbreaks.

According age–seroprevalence profiles in our study are 1: With increase in men’s age, immunity against measles also rises and 2. Compared to other investigated age groups, individuals in the 15-29 yr age group had the least immunity against measles. Concordance with our results, in Urmia of Iran and Turkey, respectively, anti-measles antibody titer was increased with the increase of age^15,18^. The reason for higher level of immunity in the higher age groups can be due to the efficacy of mass campaign for vaccination of MR. Of course, the level of measles immunity of 2-5 yr-olds prior was measured to the MR campaign, increasing age was associated with increase in antibody level against measles ^19^. Therefore the strongest hypothesis regarding this direct correlation, most likely due to boosting effect from repeated exposure to circulating wild viruses, especially in males, resulting in unapparent or sub-clinical re-infection.

In the present study, logistic regression result showed that participations in 15-29 yr had the lowest odds of measles immunity compared other retrieved age groups. In line of our finding, the level of measles immunity in this age group were lower than other age groups ^20-23^.

However, our study had some limitations: First, we could not get precise information regarding the measles vaccination date in adults in order to calculate the time interval between measles vaccination and the time to measure antibody levels for determine the effect of time on immunity level. Secondly, we also did not find information on some influential variables such as number of household members as well as being native or immigrant and travel history.

## Conclusion

Measles immunity in communities is not sufficient to prevent outbreaks and small epidemics, and periodically, serological assessments carried out at community level and especially at high risk groups. WHO certificate regarding measles elimination has been issued for Iran in 2019, maintaining the status quo requires the efforts of the country's health authorities to provide the appropriate vaccine, correct distribution, complete coverage and monitoring of the serological status of the community.

## Acknowledgements

Deputy of Research and Technology of Hamadan University of Medical Sciences approved this study (Research code: 9708014558, Ethic code: IR.UMSHA.REC.1397.485). We would like gratefully to acknowledge the health staff of Hamadan Deputy of Healths and Bahar Health Center as well as study participants.

## Conflict of interest

The author claimed no conflict of interest.

## Funding

Hamadan University of Medical Sciences financially supported this study.

## Highlights

- The overall prevalence of measles-susceptible population was considerable in the investigated population (24.6%).
- Measles immunity in communities is not sufficient to prevent outbreaks and small epidemics.
- Serological assessments at community level, especially at high-risk groups, is requires for monitor and assure of maintaining measles elimination status in Iran.
- There was no significant difference between the immunity status of the subjects in regards of their gender and age group.

## References

[1] Takahashi S, Metcalf CJE, Ferrari MJ, Moss WJ, Truelove SA, Tatem AJ (2015). Reduced vaccination and the risk of measles and other childhood infections post-Ebola. Science.

[2] World Health Organization. Measles: Key facts 2019 [updated Dec 2019; cited Dec 2019. Available from: https://www.who.int/news-room/fact-sheets/detail/measles.

[3] Chalmers I (2002). Why we need to know whether prophylactic antibiotics can reduce measles-related morbidity. Pediatrics.

[4] (2012). Centers for Disease Control and Prevention. Measles-United States. MMWR Morb Mortal Wkly Rep.

[5] He H, Chen E-f, Li Q, Wang Z, Yan R, Fu J (2013). Waning immunity to measles in young adults and booster effects of revaccination in secondary school students. Vaccine.

[6] Liu Y, Lu P, Hu Y, Wang Z, Deng X, Ma F (2013). Cross-sectional surveys of measles antibodies in the Jiangsu Province of China from 2008 to 2010: the effect of high coverage with two doses of measles vaccine among children. PLoS One.

[7] Fu C, Xu J, Liu W, Zhang W, Wang M, Nie J (2010). Low measles seropositivity rate among children and young adults: a sero-epidemiological study in southern China in 2008. Vaccine.

[8] Techasena W, Wongwacharapiboon P, Terawanich S, Pattamadilok S (2011). A comparison study of measles antibody between two doses vaccination at 9, 18 months and single dose at 9 months in children 4-6 years old. J Med Assoc Thai.

[9] Sheikh S, Ali A, Zaidi AK, Agha A, Khowaja A, Allana S (2011). Measles susceptibility in children in Karachi, Pakistan. Vaccine.

[10] Paxton GA, Rice J, Davie G, Carapetis JR, Skull SA (2011). East African immigrant children in Australia have poor immunisation coverage. J Paediatr Child Health.

[11] Poethko-Müller C, Mankertz A (2011). Sero-epidemiology of measles-specific IgG antibodies and predictive factors for low or missing titres in a German population-based cross-sectional study in children and adolescents (KiGGS). Vaccine.

[12] Poorolajal J, Khazaei S, Kousehlou Z, Bathaei S, Zahiri A (2012). Delayed vaccination and related predictors among infants. Iran J Public Health.

[13] Kang HJ, Han YW, Kim SJ, Kim Y-J, Kim A-R, Kim JA (2017). An increasing, potentially measles-susceptible population over time after vaccination in Korea. Vaccine.

[14] Izadi S, Azad TM, Zahraei S (2015). Measles vaccination coverage and seroprevalence of anti-measles antibody in south-east Islamic Republic of Iran. East Mediterr Health J.

[15] Shakurnia A, Alavi SM, Norouzirad R, Sarajian AA, Shakerinejad G (2013). Post-vaccination immunity against measles in under twenty-five-year-old population of Ahvaz, southwest of Iran. Jundishapur J Microbiol.

[16] Rasool M, Saqalein M, Saeed T, Zahoor M, Najeeb M, Siddique A (2016). Sero-epidemiology of measles in children from district Faisalabad Pakistan. Pak J Sci.

[17] Dimech W, Mulders MN (2016). A 16-year review of seroprevalence studies on measles and rubella. Vaccine.

[18] Gozalan A, Korukluoglu G, Kurtoglu D, Miyamura K, Yilmaz N, Morita M (2005). Measles seroepidemiology in 3 cities in Turkey. Saudi Med J.

[19] Yekta Z, Pourali R, Taravati M, Khalili F, Shahabi S, Salari S (2007). Measles IgG sero-prevalence and its attributable factors in 5–25-year-old cases prior mass vaccination campaign in Urmia, northeastern Iran. Iran Red Crescent Med J.

[20] Argüelles MH, Orellana ML, Castello AA, Villegas GA, Masini M, Belizan AL (2006). Measles virus-specific antibody levels in individuals in Argentina who received a one-dose vaccine. J Clin Microbiol.

[21] Jaber S (2006). A serological survey of measles, mumps and rubella immunity among school aged children in Western Saudi Arabia. Saudi Med J.

[22] Pinquier D, Gagneur A, Aubert M, Brissaud O, Le Guen CG, Hau-Rainsard I (2007). Distribution of serum measles-neutralizing antibodies according to age in women of childbearing age in France in 2005–2006: impact of routine immunization. Pediatr Infect Dis J.

[23] Wang Z, Yan R, He H, Li Q, Chen G, Yang S (2014). Difficulties in eliminating measles and controlling rubella and mumps: a cross-sectional study of a first measles and rubella vaccination and a second measles, mumps, and rubella vaccination. PLoS One.

